# Flexible Semiconductor Technologies with Nanoholes-Provided High Areal Coverages and Their Application in Plasmonic-Enhanced Thin Film Photovoltaics

**DOI:** 10.1038/s41598-017-13655-y

**Published:** 2017-10-13

**Authors:** Zhaozhao Wang, Linfa Peng, Zhongqin Lin, Jun Ni, Peiyun Yi, Xinmin Lai, Xiaolong He, Zeyu Lei

**Affiliations:** 1 State Key Laboratory of Mechanical System and Vibration, Department of Mechanical Engineering, Shanghai Jiao Tong University, Shanghai, 200240 P.R. China; 2 Department of Mechanical Engineering, University of Michigan, Ann Arbor, MI 48109-2125 USA; 3 University of Michigan-Shanghai Jiao Tong University Joint Institute, National Key Laboratory of Nano/Micro Fabrication Technology, Shanghai Jiao Tong University, Shanghai, 200240 P.R. China

## Abstract

Mechanical flexibility and advanced light management have gained great attentions in designing high performance, flexible thin film photovoltaics for the realization of building-integrated optoelectronic devices and portable energy sources. This study develops a soft thermal nanoimprint process for fabricating nanostructure decorated substrates integrated with amorphous silicon solar cells. Amorphous silicon (a-Si:H) solar cells have been constructed on nanoholes array textured polyimide (PI) substrates. It has been demonstrated that the nanostructures not only are beneficial to the mechanical flexibility improvement but also contribute to sunlight harvesting enhancement. The a-Si:H solar cells constructed on such nanopatterned substrates possess broadband-enhanced light absorption, high quantum efficiency and desirable power conversion efficiency (PCE) and still experience minimal PCE loss even bending around 180°. The PCE performance without antireflection coatings increases to 7.70% and it improves 40% compared with the planar devices. Although the advantages and feasibility of the schemes are demonstrated only in the application of a-Si:H solar cells, the ideas are able to extend to applications of other thin film photovoltaics and semiconductor devices.

## Introduction

Photovoltaic (PV) cells, which effectively convert sunlight into clean electrical power, provide virtually unlimited amounts of renewable energy. Silicon has been the material of choice for PV cells owing to low cost, earth abundance, non-toxicity, and the availability of mature processing technologies^1^. However, the cost of current PV devices still needs to be substantially reduced so that large scale implementation of PV modules could be realized. Thin-film, second-generation solar cells of CdTe, amorphous Si, and CuInxGa1-xSe2 (CIGS) fabricated with absorbing layers of a few micrometers thick may provide a viable way towards this goal because of their small material assumption and low processing costs^2–4^. In addition, thin-film solar cells could be built to form mechanically flexible or even stretchable optoelectronic systems with inorganic materials. Some concepts integrating inorganic materials with organic substrates are now available for fabricating optoelectronic systems possessing a combinational effect of the mechanical robustness and superior light trapping^5,6^.

Resent research exploits ways to build inorganic photovoltaic (PV) systems on flexible substrates, which could integrate excellent optoelectronic performances with mechanical flexibility. Compared to schemes built on planar, rigid substrates, such schemes enable various reversible deforming modes of inorganic optoelectronic systems, including bending, stretching, compressing or twisting, thereby expanding the potential applications from building-integrated optoelectronic devices to portable energy sources. There are a variety of choices of flexible substrates for flexible PV systems, such as metallic foils (Ti foils, Al foils, etc), thin glasses and membrane plastics^7–10^. With superior mechanical robustness and lightweight characteristics, plastic nano/micro membrane materials are now available in wearable electronics, flexible display, thin film solar cells and many others. Meanwhile, difficulty in improving light harvesting capability could be addressed by using nanostructure-decorated plastic membrane materials for flexible photovoltaic applications. A number of three-dimensional nanostructures, such as nanopillars, nanopyramids, nanowells, nanorods, nanovoids and nanowires, have been systematically studied to improve the light absorption ability of thin film solar cells^11–13^. Typically, metallic nanostructures have been adopted as front or back electrodes of solar cells owing to the plasmonic effects^14–21^. Plasmonic metallic nanostructures result in light scattering and tend to enable unparalleled light concentration and trapping in the absorption layer^14,15,22–28^.

In our work, hydrogenated amorphous silicon (a-Si:H) solar cells have been constructed on nanotextured polyimide (PI) films, on which the nanostructures are constructed by a soft thermal nanoimprinting method using inorganically cross-linked TiO_2_ sol-gels. The imprinted inorganic nanostructures, on which various thin film solar cells could be constructed, possess outstanding thermal and mechanical reliability compared to conventional organic resists. The sol-gel based nanoimprinting method is developed to achieve regular nanohole structures without relying on complicated and costly lithographic techniques. The results demonstrate the compatibility with thin, brittle organic semiconductor devices. The outstanding mechanical and optoelectronic performances demonstrated by a-Si:H solar cells improve the chances for realistic use of such nanotextured plastic substrates.

## Experimental

Preparation of TiO_2_ sol: 1 M TiO_2_ sol was prepared by mixing together 0.1 mol of Titanium isopropoxide (TiPP) as a precursor in 0.1 L of ethanol absolute as the solvent medium and dropping diethanolamine (DEA) into the solution as a sol stabilizer while maintaining the molar ratio of DEA/Ti at 1:1, then stirring until completely melted^21^.

**Figure 1:**
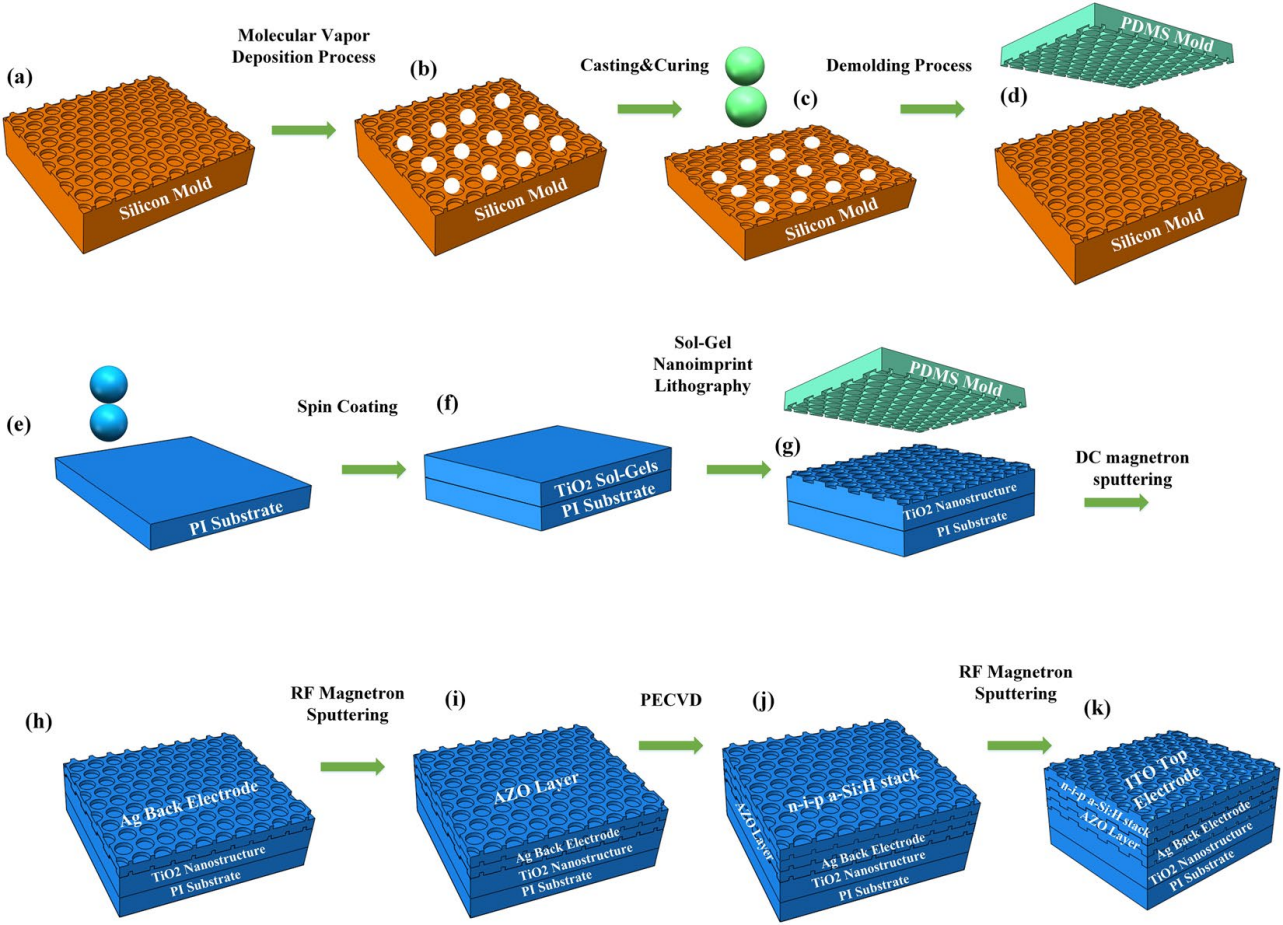
Schematic illustration of fabrication of PDMS stamps and a-Si:H solar cells on nanopatterned plastic substrates: (**a**) Si master stamp, (**b**) An anti-sticking coating on Si master stamp by a molecular vapor deposition process, (**c**) PDMS precursor casted onto the anti-sticking surface of the Si master stamp, (**d**) PDMS soft mold separated from the Si master stamp, (**e**) Pre-cleaned PI film, (**f**) TiO_2_ sol film by spin coating on the PI substrate, (**g**) Textured TiO_2_ sol film on the PI substrate via soft thermal nanoimprint process, (**h**) Ag back electrodes deposited on TiO_2_ nanostructures by DC magnetron sputtering, (**i**) AZO spacer layer deposited on the Ag bottom, (**j**) n-i-p a-Si:H stacks constructed on top of the AZO layer, (**k**) the ITO top electrode deposited on amorphous silicon layer.

Preparation of nanotextured TiO_2_/PI film by soft thermal nanoimprinting lithography: Surface-textured TiO_2_ films were obtained by direct imprinting TiO_2_ sol on PI films using polydimethylsiloxane (PDMS) soft molds and subsequent annealing process^29^. The PDMS soft molds with hexagonally packed nanopillar arrays (pitch size = 1 µm, pillar diameter = 800 nm, pillar height = 400 nm) were produced by casting PDMS liquids into Si master stamp and subsequent baking process. An anti-sticking fluorinated silane precursor was deposited onto the Si master mold via a molecular vapor deposition process (Sylgard) under 100 °C. The PDMS (Sylgard 184) stamps were produced by casting a 10:1 weight ratio mixture of basic agent and firming agent onto the Si mold and then curing at 60 °C for 3 h. The process for the fabrication of the PDMS stamp was illustrated in Fig. 1a–d.

The TiO_2_ sol was spin coated on a PI substrate (UBE Industries, Ltd., 25 m) at 3000 rpm for 50 s. The textured PDMS mold was then attached onto the TiO_2_ sol membrane and located into a nanoimprinting chamber (NC-AX1401, Swind Ltd.). A soft thermal nanoimprinting process was conducted under 0.6 MPa and 250 °C for 2 h until the nanotextured TiO_2_-gels was completely formed. After the soft thermal imprinting process the PDMS mold was peeled off from the surface of the nanotextured TiO_2_-gels and the TiO_2_-gels on PI substrates were further cured in air at 250 °C for 2 h to remove the solvent residues. The soft thermal nanoimprinting process for fabrication of nanotextured TiO_2_/PI film was illustrated in Fig. 1e–g.

Fabrication of a-Si:H solar cells: A Ag reflector layer with the thickness of 100 nm was deposited on the obtained nanotextured PI substrate by direct current (DC) magnetron sputtering in argon plasma atmosphere at room temperature. A 2 wt% Al_2_O_3_ doped ZnO ceramic (99.9% purity) layer was sputtered by radio frequency (RF) magnetron sputtering under argon plasma at the temperature of 250 °C. Subsequently, a stack of n-i-p amorphous silicon layers were successively deposited in a PECVD multi-chamber system composed of three PECVD chambers under 250 °C. The n-doped and p-doped layers have the thickness of 30 nm and 10 nm, respectively, while the intrinsic layer has the thickness of 280 nm. After the fabrication of amorphous silicon layers, an ITO layer with the thickness of 80 nm was deposited by RF sputtering as the top electrode. Finally, a Ag grid was thermally evaporated over ITO as the contact electrode via a contact mask. The same fabrication processes were also conducted on a flat PI substrate to fabricate the flat amorphous silicon solar cell for comparison. The fabrication process of a-Si:H solar cells was presented in Fig. 1h–k.

FEM & electromagnetic simulations: ABAQUS commercial software was employed to study the mechanical response of the nanohole structures. The polymeric PI substrate was modeled by the hexahedron element (C3D8R), while the nanotextured stiff thin TiO_2_ films were modeled by the composite shell element (S4R). The electromagnetic mechanism was analyzed by using Lumerical FDTD solutions. A plane wave light source irradiated normally to the devices. The source was set to be polarized along the x-axis considering that the hexagonally arranged structure is polarization independent. The representative cell of the patterned structure was set as the simulation region using anti-symmetrical boundaries in the x-axis, symmetrical boundaries in the y-axis and PML boundaries in the z-axis. Complex refractive indices of Ag were adopted from Palik’s handbook of Optical Constants while those of AZO, ITO and n-i-p a-Si:H were modelled using the measured data. Those measured refractive indices and extinction coefficients were plotted in Fig. S3.

Device characterization: SEM images of the a-Si:H PV structures were taken by JEOL JSM-7800F Prime(SEM)&Thermo ScientificTM NORANTM Syst. All the J-V curves of a-Si:H solar cells were carried out using a solar simulator (Newport corporation, 91150 V) under 1 sun illumination. The EQE measurements were characterized by Oriel QE-PV-SI (Newport Corporation). Mechanical properties of all samples were measured with a dynamic mechanical analyzer (TA instruments, Q800). Each of the reported results corresponded to an average of measurements on three samples.

## Results and Discussion

### Mechanical Characterization

**Figure 2:**
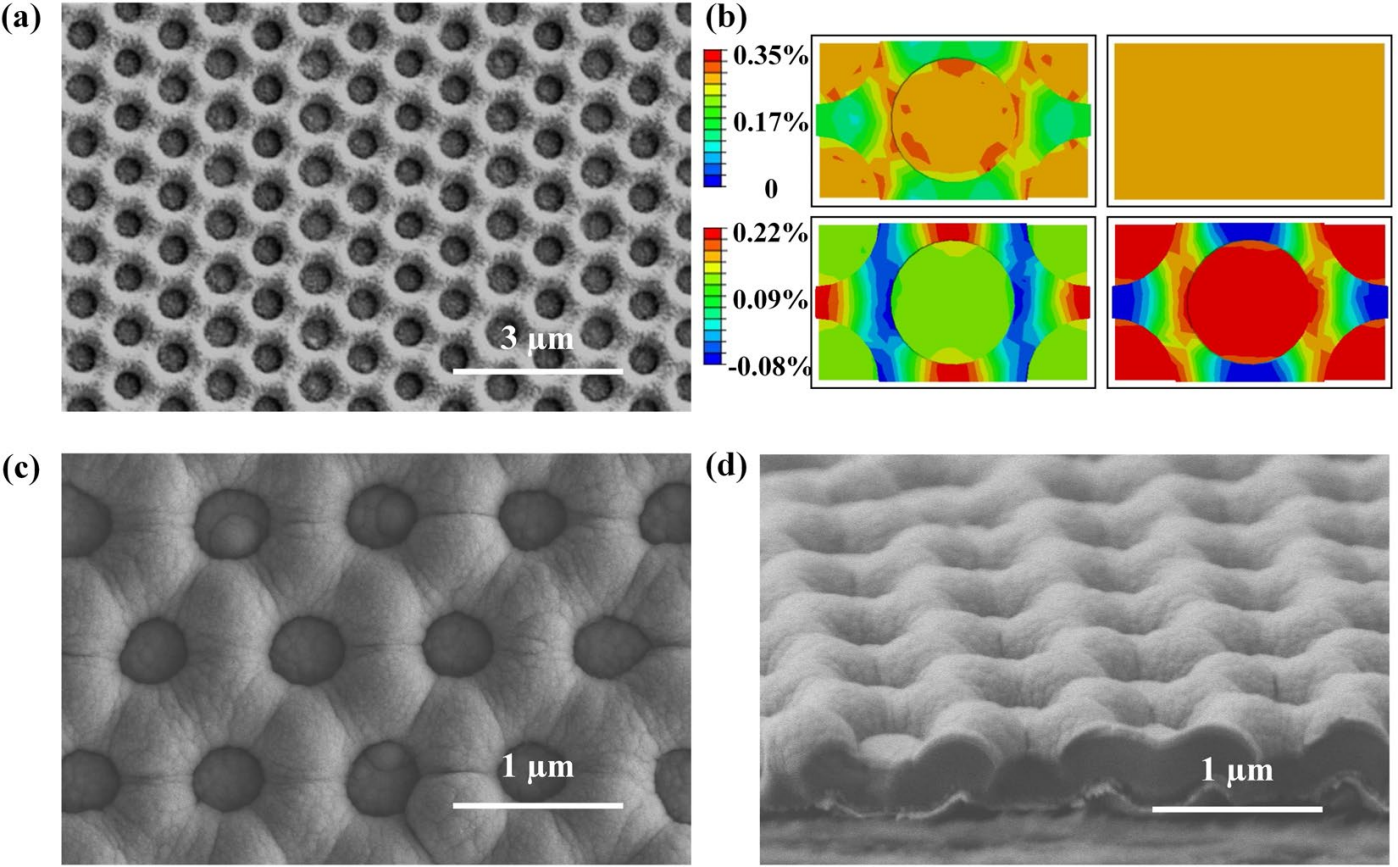
(**a**) Scanning electron microscopy (SEM) images of a slab of PI film with patterned surface in the geometry of hexagonally ordered nanohole arrays; (**b**) Finite element modeling (FEM) of this structure in a biaxially stretched state, FEM results of the distribution of maximum principal strain and the two components of the strain tensor at the top surface of the nanoholes decorated substrates; (**c**) Top and (**d**) Cross sectional scanning electron microscopy (SEM) images of a-Si:H solar cells constructed on the patterned substrate.

Structure decorated plastic substrates, as shown in Fig. 2a, involve hexagonally ordered arrays of nano-holes (NHs) with the pitch of 1 µm and aspect ratio of 0.25. The diameter of the nano-hole (NH) is 800 nm. The average coverage of the nano-hole arrays corresponds to 160.5% of the overall flat area. Fig. 2b shows FEM results of the distribution of maximum principal strain and the two components of the strain tensor at the top surface of the representative cell for nano-hole arrays in a biaxially stretched state. The small thickness at the nano-hole regions reduces the cross sectional area, thereby leading to the strain localization to these regions when deforming the overall system. Furthermore, the strains decrease from the nano-holes’ base areas to the top surfaces, resulting in minimal strain at the locations supporting the organic materials compared with the planar devices.

Fig. S1a shows bending a structured substrate with the curvature radius of 100 µm. The regions near the bottom of nano-holes absorb the bending strains, while keeping the top surface with minimal strains. The strain computed by FEM is 0.12% at the top surface. Due to the presence of nano-holes, the system’s breakage would be prevented from excessive strains. This results correspond well with the experimental bending test results in which the power conversion efficiency of the patterned solar cells only drops 16.5% after bending at the angle of 180°.

A substrate under applied uniaxial strain of 1.0%, which doesn’t exceed fracture limits of organic materials, appears in Fig. S1b. The substrates elongate by 0.9% approximately along the direction of stretching and they contract by 0.5% approximately in the orthogonal direction due to Poisson effect. Whereas the flat substrate only elongate by 0.7% under the same applied uniaxial strain. The structured substrates improve the overall stretchability by approximately 30% compared to the flat substrates, and the stretchability could be further improved by trenches structures and appropriate selection of trenches’ heights^30^.

In addition to outstanding mechanical behaviors and high areal coverages provided by the nano-hole arrays, the structured substrate has the added advantage of making the plasmonic substrate in order to construct optoelectronic devices on it with superior optoelectronic performances. Amorphous silicon solar cells have been constructed on the hexagonally ordered arrays of nano-holes decorated PI substrates as the module demonstration. Fig. 2c,d show present top and cross sectional views of a-Si:H solar cells fabricated on the nanopatterned substrates. The photographs of solar cells on the nanopatterned and flat substrates are presented in Fig. S2.

### Optical Characterization

**Figure 3:**
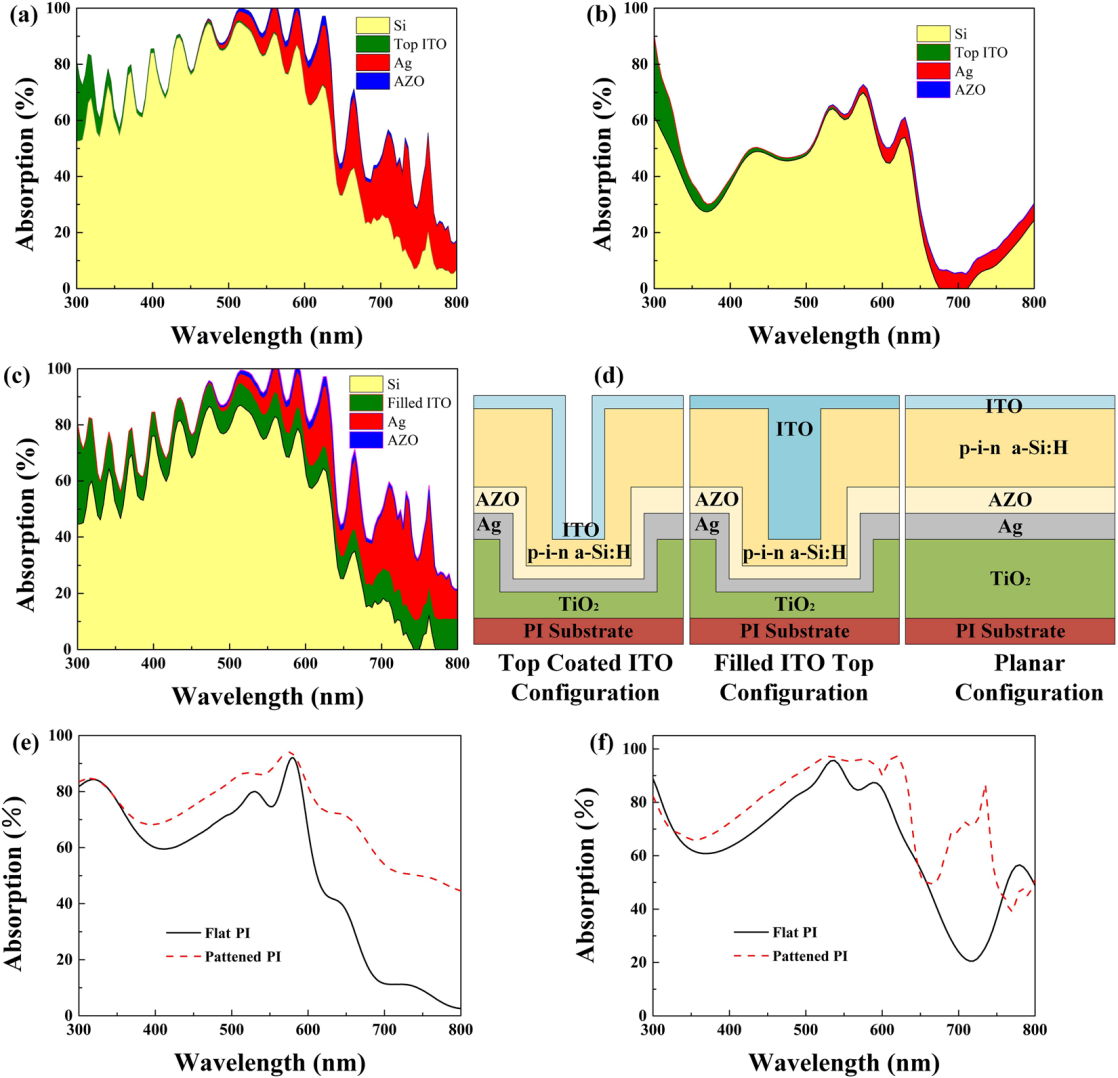
(**a**) Plots of calculated absorption in each layer of the patterned devices (Top coated ITO configuration); (**b**) Plots of calculated absorption in each layer of the planar devices (Planar configuration); (**c**) Plots of calculated absorption in each layer of the patterned devices (Filled ITO top configuration); (**d**) Schematic structures of fabricated solar cells, Top coated ITO configuration, Filled ITO top configuration and Planar configuration, respectively; (**e**) Experimental absorption spectra of the a-Si:H solar cells on the planar and patterned PI films; (**f**) Simulated absorption spectra of the a-Si:H solar cells on the planar and patterned PI films.

The Ag layer, whether supported by a flat PI film or a nanopatterned PI film, plays a role of the back reflector layer and the bottom electrode, while the AZO layer functions as a buffer layer preventing the metal diffusion into the amorphous silicon layer and reducing the undesired light absorption in the Ag bottom electrode caused by the excitations of surface plasmons^31^. The definition of the nanohole’s normalized thickness $\bar{t}$ is the depth divided by the diameter. The nanopatterned device with the normalized thickness $\bar{t}$ of 0.25 has a higher light capturing capability than the flat counterpart demonstrated by broadband absorption. The ITO top layer is 80 nm thick, which approximately gives the minimum reflectance as reported. Although ITO electrodes give the parasitic light absorption compared to bare silicon NH arrays in terms of optical characterization, the electrical behavior of ITO can play an important role in electron transport. In addition, the top configuration of ITO coating layer causes minimum parasitic light absorption compared to other ITO electrode configurations such as filled ITO top layer configurations as shown in Fig. 3a and Fig. 3c. Therefore, the top coated ITO configuration based on NH arrays electrode has been adopted owing to the added advantage of fast electron transport.

**Figure 4:**
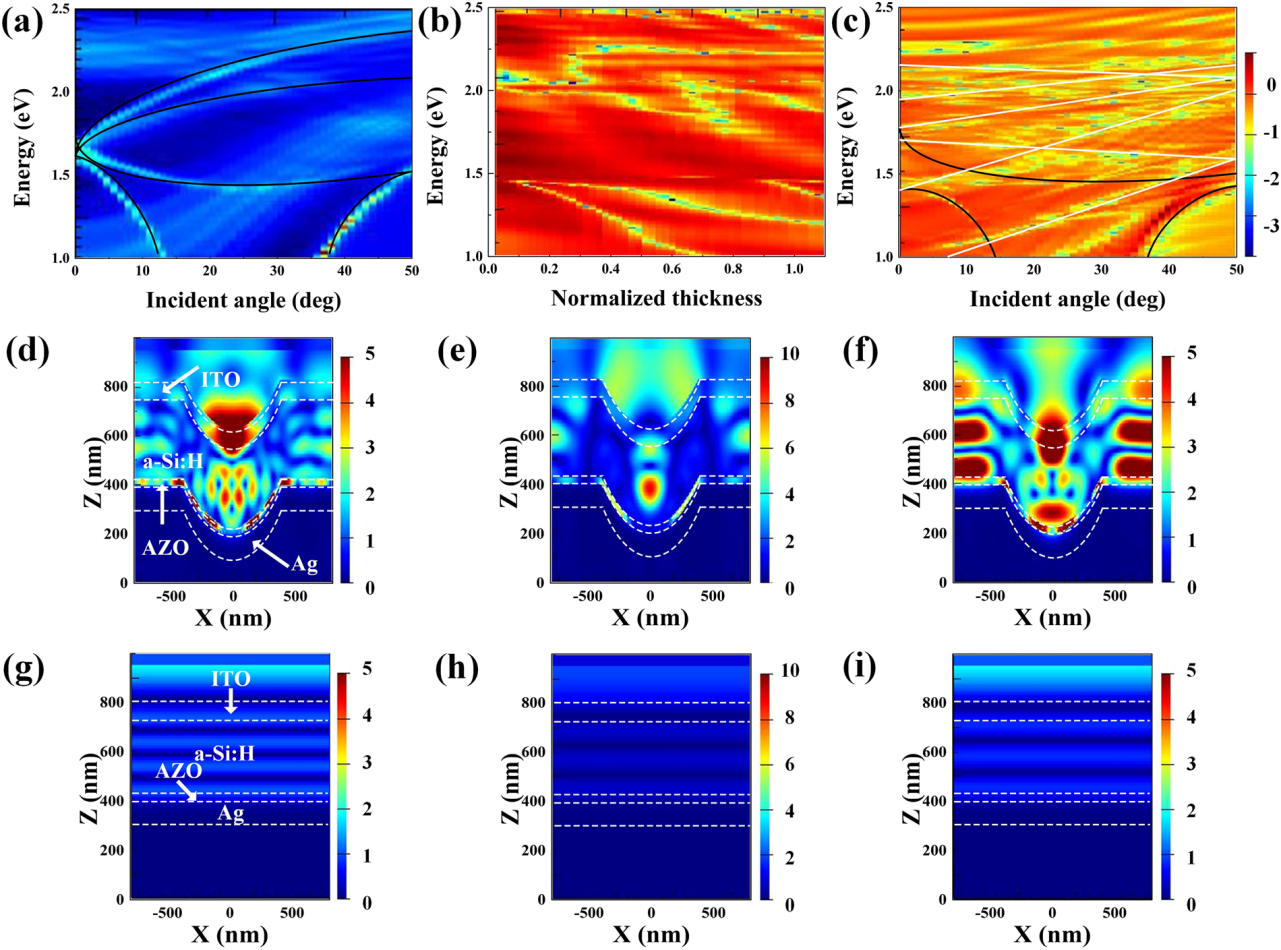
(**a**) Dispersion plots of samples at $\bar{t}=0.25$ and $\varphi =0$ with nanohole diameter of 800 nm at different photo energies and incident angles. Lines show theory for Bragg plasmons; (**b**) Mie plasmon absorption for samples with nanohole diameter of 800 nm, showing plasmon mode energy vs t at normal incidence; (**c**) Angle reflectance scans of complete solar cells as indicated. Color scale is log(reflectance) with red representing high reflectance and blue-white representing low reflectance; (**d**–**i**) The cross-sectional views of electric field intensity (E) distributions in patterned devices and planar devices at wavelengths of 733 nm, 860 nm and 916 nm.

The NH array exhibits a higher absorption spectrum than the nonpatterned a-Si:H over the simulated spectrum range. It could be observed that the light absorption under shorter wavelength mainly takes place in the ITO top layer as shown in Fig. 3a. The absorption enhancement seen in the short wavelength region is expected from the scattering and gradient effective refractive index induced antireflective effect. In the longer wavelength region, a strong absorption could be found in the amorphous silicon layer as demonstrated by the calculated electric field distributions in Fig. 4d and the NH array absorption spectra shows absorption peaks with a complex, irregular shape which is beneficial for approaching Yablonovitch limit as shown in Fig. 3a. The absorption enhancement in this wavelength range could be expected from diffraction trapping of incident light and coupled resonant modes attributed to the NH array. Several additional peaks caused by resonant modes can be observed in this wavelength range through a comparison with the nonpatterned a-Si:H as shown in Fig. 3b. Most of the light absorption happens in the a-Si:H layer, which is desirable for the generation of electron-hole pairs. The simulation in Fig. 3f shows more absorption peaks and a slightly lower absorption trend over the wavelength range than that seen in the measurement data as shown in Fig. 3e, this could be mainly caused by the difference in the incident and detected angles in the measurement and simulation. In addition, defects such as roughness and other undesirable film nonuniformity could not be avoided in fabrication and these could be responsible for a compensation of the rapid fluctuations.

The influence of surface plasmon polaritons (SPPs) on the optical performance of solar cells has been investigated in many studies. Surface plasmon polaritons (SPPs) are termed as infrared or visible-frequency electromagnetic waves that travel along a metal-dielectric or metal-air interface^32,33^. They are expected to improve absorption by confining the coupled electromagnetic energy at the metal-semiconductor interface and propagating laterally. The integration of metallic nanostructures that support SPP into thin layer solar cells has been proposed as a way to enhance light trapping and absorption. In this study metallic NH arrays are employed to produce propagating surface plasmon waves for boosting the efficiency of amorphous silicon solar cells. To study the surface plasmons alone, the spectral dispersion of several different types of surface plasmon modes is investigated by recording the reflectivity spectrum of the NH array metallic structure for different incident angles as shown in Fig. 4a. Plasmons propagating in different directions have different energies at the same incident angle in these dispersion relations even though the SPP dispersion is isotropic attributed to the geometry of the optical interaction. In this study, most of SPPs are termed as “Bragg plasmons” due to their coupling via Bragg scattering so that they could be distinguished from other plasmon oscillations^34^. Generally Bragg plasmons are excited in six in-plane directions at six different energies. However, degeneracies at symmetry orientations reduce the number of modes to 4. The bottom right line shows the plasmon band excited by the next-nearest wave vectors. Fig. 4b shows Mie plasmon mode energy for samples with nanohole diameter of 800 nm with respect to normalized thickness of nanoholes at normal incidence. For $\bar{t} < 0.5$ the mixing with Bragg plasmon modes results in a more complex dispersion. The Mie plasmons can be clearly identified for $\bar{t} > 0.5$. The energy of Bragg modes varies as the incident and azimuthal angles $\varphi$ are varied at any point on the sample while the Mie plasmon energies could be controlled via changing the normalized thickness of the nanoholes. This gives availability to bring these two types of plasmons into resonance which is in favor of light energy confinement in solar cells.

Because of the coverage of the waveguide modes, most SPP modes can hardly be observed directly. Electric field distribution at a wavelength of 733 nm in Fig. 4d presents the bounded energy at the surface of the Ag back reflector by SPP mode. Dispersions of localized surface plasmon (LSP) resonance and waveguide modes still could be observed, implying that they lead to obvious absorption enhancement in a specific wavelength region. At the tips of nanoholes, there exists a strong near field absorption as shown in Fig. 4d. In spite of the poor absorption ability of a-Si:H in this wavelength region, more effective absorption enhancement is expected to be realized by tuning LSP resonance frequency to the visible light region through changing the size, shape, plasmonic materials and surrounding dielectrics of triangular pyramid tips^32–39^. The reflected electromagnetic (EM) waves from sidewalls of nanoholes yield converge at a location inside the nanohole. Fig. 4e illustrates the effective optical coupling between the reflected EM waves and SPPs leading to strong absorption. The results here indicate that plasmon resonances are still beneficial for silicon layer absorption. Bragg scattering-like surface plasmon modes indicated in black lines in Fig. 4c alongside photonic or waveguide modes in white lines arising from the hexagonal lattice of nanoholes are present in the amorphous silicon solar cell with a thin AZO coating. The greatest contribution to the absorption enhancement comes from the coupling of several waveguide modes excited within the patterned multi-layer structures. The SPP mode is bound to the semiconductor-Ag interface, while the waveguide modes are localized mainly in the amorphous silicon layer. For instance, at the wavelength of 916 nm, most of the energy is confined within the silicon layer by effective optical coupling between the incident light and the large density of waveguide modes as shown in Fig. 4f.

The amorphous silicon layer corresponds to the active waveguide layer, while the Ag back electrode represents the cladding layer. The AZO layer and the ITO layer act as low index spacers. Niraj N. Lal has pointed out the main effect of localized plasmons in silver nanoholes with 30 nm of spacer layers to be quenching of optical modes leading to parasitic absorption in the metal^40^. The AZO buffer layer between the amorphous silicon layer and the Ag back reflector is beneficial for effective light absorption because it converts the p-polarized zero order mode from the excitation of a surface plasmon resonance localized at the metallic interface into a regular waveguide mode whose field intensity is located mainly in the amorphous silicon film^31^. Therefore, parasitic absorption in the metal film is reduced and more absorption takes place in the silicon layer which is favorable to the power conversion enhancement of the PV devices. In-depth investigation on optimizing spacer thickness is highly desired for further performance enhancement.

For comparison, electric field distributions of flat cells are also illustrated in Fig. 4g–i . Without nanostructured substrate induced SPPs, LSPs and waveguide modes, only interference fringes could be observed. Electric field cannot be effectively confined within the absorbing layer, and most of the incident light passes through the silicon layer and is reflected backward. Electric field profiles for flat cells show minimal overlap with the a-Si:H layer at the wavelength of 733 nm, 860 nm and 916 nm, confirming the need for scattering features to couple incident light to guided modes.

**Figure 5:**
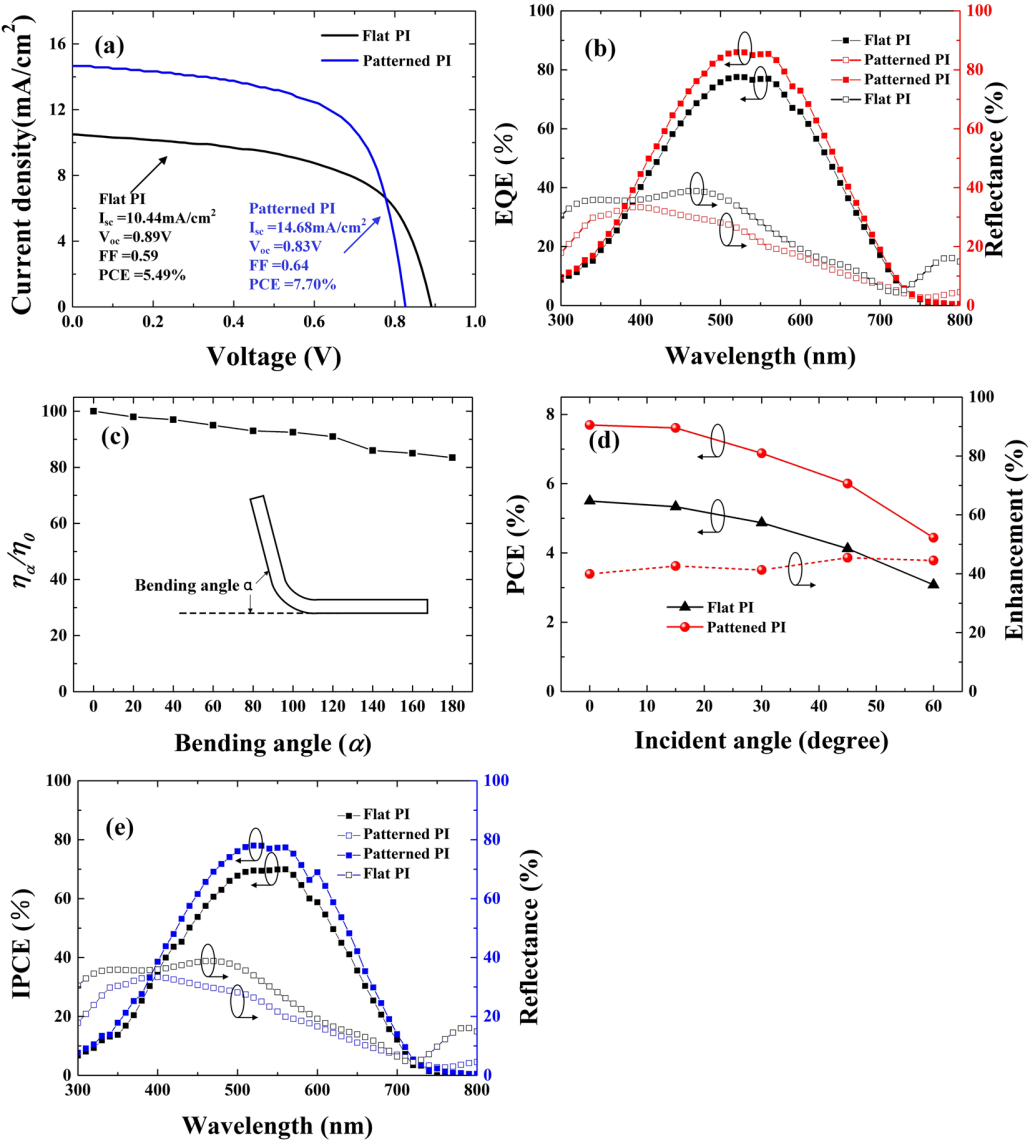
(**a**) Current-voltage characteristics. The short circuit current density (J_sc_), open circuit voltage (V_oc_), fill factor (FF), and power conversion efficiency (PCE); (**b**) External quantum efficiency (EQE) and reflectance of a-Si:H devices based on PI film with TiO_2_-gel patterns and planar substrates; (**c**) Relative PCE efficiency of the patterned devices as a function of bending angles, the inset illustrates the definition of the bending angle; (**d**) PCEs and PCE enhancement of patterned devices with respect to flat devices as a function of incident angles; (**e**) Incident Photon-Electron Conversion Efficiency spectrum (IPCE) and reflectance of a-Si:H devices based on PI film with TiO2-gel patterns and planar substrates.

### Device Characterization

The above optical investigation shows the potential of NH structures for efficient photon capturing. Meanwhile, the device performance of the NH based thin film a-Si:H solar cells (without antireflection coatings) is characterized together with the flat reference device. Fig. 5a presents current density-voltage (J-V) characteristics of the two kinds of devices discussed above with a solar simulator (Newport Corporation, 91150 V) under 1 sun illumination. J_sc_ and V_oc_ extracted from these J-V curves are summarized in Fig. 5a. The J_sc_ increases from 10.44 mA/cm^2^ to 14.68 mA/cm^2^ using nanopatterned TiO_2_/PI films instead of the flat PI substrates, attributed to the improved light absorption capability. The increased J_sc_ of the NH device can be confirmed with external quantum efficiency (EQE) measurements as shown in Fig. 5b, which presents a broadband spectra response enhancement with respect to the planar reference device. The fill factor (FF) is 0.64 and the improved FF mainly results from the advantage of U-shape geometry of the patterned NH structure. Since the reflectance of the NH patterned device is ∼22% for the incident light, we expect that the power conversion efficiency (PCE) can be improved to ∼40%. Although V_oc_ is slightly decreased owing to the texture induced defects on the patterned substrate, the overall PCE performance of NH device improves from 5.49% to 7.70% attributed to the enhanced optical absorption and increased photocurrent. In addition, it is known that the direction of solar irradiation varies over time in a day. Therefore, the study of irradiation-dependent PCE has great importance to the PV community. Fig. 5d shows the PCEs of NH and flat reference devices for the light incident angles tuning from 0° (normal incident) to 60° with a 10° interval, where the PCE values are calculated using the device active area, regardless of the change of projection area under different incident angles. It is clearly seen that the NH devices possess higher PCEs over all incident angles with an enhancement of ∼40% compared to the flat counterpart. These results show the outstanding significance for practical employment of patterned PI/TiO_2_ substrates in light management of thin film solar cells. Fig. 5e shows the IPCE spectrums with the AM 1.5 G light illumination in the devices with and without the patterned substrates. As compared to the less than 90% absorption in the absorption spectrum of the patterned devices, the around 75% IPCE implies the effectiveness of carrier extraction. For the planar devices the IPCE is around 68% under the absorption of 85%.

The PI material, which possesses better flexibility than metal foils and a higher temperature tolerance than polyethylene terephthalate (PET) and polyethylene naphthalate (PEN), is expected to be a promising choice for flexible electronics. To evaluate the robustness of the PI-film-based device in this work, the PCEs of the nanopatterned solar cells under a bending angle from 0° to 180° are characterized with a homemade setup. The normalized efficiencies are calculated using the device projection area. It is found that the device only experiences about 16.5% efficiency reduction even under 180° bending angle, as shown in Fig. 5c. Moreover, even after 100,000 bending cycles, there are no remarkable reduction. It is known that the a-Si:H possesses excellent mechanical robustness and has been widely employed in flexible devices. However, the metal oxides, including AZO and indium-doped tin oxide (ITO) are ceramic materials, which are brittle and may decrease the device flexibility. It has been found that a thinner ITO yields a decreased internal stress and an increased crack onset strain. For instance, the crack onset strain of ITO films increases from 1.52% to 2.19% as the thickness decreases from 100 to 50 nm^41^. In our work, the thicknesses of AZO and ITO are 30 and 80 nm, respectively and the reduced thickness around several nanometers may depress the internal brittleness. Moreover, the outstanding flexibility could dominantly benefit from the preformed TiO_2_ NH arrays on PI substrate, which provides a larger surface area in the interfacial regions between each layer and in turn induces lower stress distributed across all functional layers with more uniformness. The concepts reported here have the potential to be useful for a range of other applications, owing to their applicability to many classes of semiconductor devices, from light emitting diode displays/lighting systems to digital imagers, integrated circuits and others.

## Conclusions

In summary, in this work, we demonstrated highly ordered metal oxide patterns on flexible PI foils by a cost-effective sol-gel soft imprint process, which efficiently suppressed the internal stress during the a-Si:H solar cell fabrication and in turn is beneficial for the mechanical robustness of the device. The remarkable and broadband enhancements in optical absorption and quantum efficiency were realized on NH device with nanohole array back reflector attributed to the hybrid optical modes excited by nanotextured back reflector. Moreover, the flexibility has been also considered as a function of bending angle and bending cycle. The nanopatterned TiO_2_/PI substrate delivered an excellent device performance without performance degradation even after 100,000 bending cycles, owing to the suppressed stress across functional layers. Although the work was conducted on a-Si:H material, our proposed scheme can be extended to a variety of active materials for optoelectronics application. For future work, the UV-curable inorganic oxide-organic composites and roll-to-roll process will be employed for more efficient production of photonic structures and antireflective moth-eye structures will be adopted as a top coverage layer.

### Data availability statement

The datasets generated during and/or analyzed during the current study are available from the corresponding author on reasonable request.

## Electronic supplementary material

Supplementary information

## References

[1] Green MA (2004). Recent developments in photovoltaics[J]. Sol. energy.

[2] Chirilă A, Buecheler S, Pianezzi F (2011). Highly efficient Cu (In, Ga) Se2 solar cells grown on flexible polymer films[J]. Nat. Mater..

[3] Liu D, Kelly TL (2014). Perovskite solar cells with a planar heterojunction structure prepared using room-temperature solution processing techniques[J]. Nat. Photonics.

[4] Tan H, Furlan A, Li W (2016). Highly efficient hybrid polymer and amorphous silicon multijunction solar cells with effective optical management[J]. Adv. Mater..

[5] Yang J, Banerjee A, Guha S (1997). Triple-junction amorphous silicon alloy solar cell with 14.6% initial and 13.0% stable conversion efficiencies[J]. Appl. Phys. Lett..

[6] Hegedus S (2006). Thin film solar modules: the low cost, high throughput and versatile alternative to Si wafers[J]. Prog. Photovolt. Res. Appl..

[7] Yusoff ARM, Syahrul MN, Henkel K (2007). Film adhesion in amorphous silicon solar cells[J]. Bulletin of Materials Science.

[8] Kaltenbrunner M, Adam G, Głowacki ED (2015). Flexible high power-per-weight perovskite solar cells with chromium oxide-metal contacts for improved stability in air[J]. Nat. Mater..

[9] Zhang Y, Du Y, Shum C (2016). Efficiently-cooled plasmonic amorphous silicon solar cells integrated with a nano-coated heat-pipe plate[J]. Sci. Rep..

[10] Peng KQ, Wang X, Li L (2010). High-performance silicon nanohole solar cells[J]. Journal of the American Chemical Society.

[11] Wang B, Leu PW (2012). Enhanced absorption in silicon nanocone arrays for photovoltaics[J]. Nanotechnology.

[12] Kelzenberg MD, Boettcher SW, Petykiewicz JA (2010). Enhanced absorption and carrier collection in Si wire arrays for photovoltaic applications[J]. Nat. Mater..

[13] Lin YR, Lai KY, Wang HP (2010). Slope-tunable Si nanorod arrays with enhanced antireflection and self-cleaning properties[J]. Nanoscale.

[14] Ferry VE, Sweatlock LA, Pacifici D (2008). Plasmonic nanostructure design for efficient light coupling into solar cells[J]. Nano lett..

[15] Pala RA, White J, Barnard E (2009). Design of plasmonic thin-film solar cells with broadband absorption enhancements[J]. Adv. Mater..

[16] Panoiu NC, Osgood RM (2007). Enhanced optical absorption for photovoltaics via excitation of waveguide and plasmon-polariton modes[J]. Optics lett..

[17] Chen HL, Chuang SY, Lin CH (2007). Using colloidal lithography to fabricate and optimize sub-wavelength pyramidal and honeycomb structures in solar cells[J]. Opt. Express.

[18] Han SE, Chen G (2010). Toward the Lambertian limit of light trapping in thin nanostructured silicon solar cells[J]. Nano lett..

[19] Mavrokefalos A, Han SE, Yerci S (2012). Efficient light trapping in inverted nanopyramid thin crystalline silicon membranes for solar cell applications[J]. Nano lett..

[20] Lin H (2014). Rational design of inverted nanopencil arrays for cost-effective, broadband, and omnidirectional light harvesting[J]. ACS nano.

[21] Firdaus CM (2012). Characterization of ZnO and ZnO: TiO_2_ thin films prepared by sol-gel spray-spin coating technique[J]. Procedia Engineering.

[22] Hsu CM, Battaglia C, Pahud C (2012). High-Efficiency Amorphous Silicon Solar Cell on a Periodic Nanocone Back Reflector[J]. Adv. Energy Mater..

[23] Sai H, Fujiwara H, Kondo M (2008). Enhancement of light trapping in thin-film hydrogenated microcrystalline Si solar cells using back reflectors with self-ordered dimple pattern[J]. Appl. Phys. Lett..

[24] Sai H (2012). Enhanced photocurrent and conversion efficiency in thin-film microcrystalline silicon solar cells using periodically textured back reflectors with hexagonal dimple arrays. Appl. Phys. Lett..

[25] Genet C, Ebbesen TW (2007). Light in tiny holes[J]. Nature.

[26] Sai H, Fujiwara H, Kondo M (2008). Enhancement of light trapping in thin-film hydrogenated microcrystalline Si solar cells using back reflectors with self-ordered dimple pattern[J]. Appl. Phys. Lett..

[27] Petermann JH, Zielke D, Schmidt J (2012). 19%-efficient and 43 µm-thick crystalline Si solar cell from layer transfer using porous silicon[J]. Prog. Photovolt. Res. Appl..

[28] Schmidt W, Woesten B, Kalejs JP (2002). Manufacturing technology for ribbon silicon (EFG) wafers and solar cells[J]. Prog. Photovolt. Res. Appl..

[29] Peroz C, Chauveau V, Barthel E (2009). Nanoimprint Lithography on silica sol–gels: a simple route to sequential patterning[J]. Adv. Mater..

[30] Ma Y, Jang KI, Wang L (2016). Design of Strain-Limiting Substrate Materials for Stretchable and FlexibleElectronics[J]. Adv. Func. Mater..

[31] Haug FJ, Söderström T, Cubero O (2009). Influence of the ZnO buffer on the guided mode structure in Si/ZnO/Ag multilayers[J]. J. Appl. Phys..

[32] Söderström T, Haug FJ, Terrazzoni-Daudrix V (2010). Flexible micromorph tandem a-Si/μ c-Si solar cells[J]. J. Appl. Phys..

[33] Battaglia C, Hsu CM, Söderström K (2012). Light trapping in solar cells: can periodic beat random?[J]. ACS nano.

[34] Haug FJ, Söderström T, Python M (2009). Development of micromorph tandem solar cells on flexible low-cost plastic substrates[J]. Sol. Energy Mater. and Sol. Cells.

[35] Van Lare M, Lenzmann F, Verschuuren MA (2012). Mode coupling by plasmonic surface scatterers in thin-film silicon solar cells[J]. Appl. Phys. Lett..

[36] van Lare C, Lenzmann F, Verschuuren MA (2015). Dielectric scattering patterns for efficient light trapping in thin-film solar cells[J]. Nano lett..

[37] Spinelli P, Ferry VE, Van de Groep J (2012). Plasmonic light trapping in thin-film Si solar cells[J]. J. Optics.

[38] Ferry VE, Verschuuren MA, Li HBT (2010). Light trapping in ultrathin plasmonic solar cells[J]. Opt. express.

[39] Nomura K, Ohta H, Takagi A (2004). Room-temperature fabrication of transparent flexible thin-film transistors using amorphous oxide semiconductors[J]. Nature.

[40] Lal NN, Zhou H, Hawkeye M (2012). Using spacer layers to control metal and semiconductor absorption in ultrathin solar cells with plasmonic substrates[J]. Phys. Rev. B.

[41] Leterrier Y, Medico L, Demarco F (2004). Mechanical integrity of transparent conductive oxide films for flexible polymer-based displays[J]. Thin Solid Films.

