# Supplementary material for: Flexible Semiconductor Technologies with Nanoholes-Provided High Areal Coverages and Their Application in Plasmonic-Enhanced Thin Film Photovoltaics
Source: Sci Rep. 2017 Oct 13;7:13155. doi: 10.1038/s41598-017-13655-y (PMC5640606; doi:10.1038/s41598-017-13655-y)
Supplement: Supplementary file 1 — Supplementary information [file 41598_2017_13655_MOESM1_ESM.pdf]

Flexible Semiconductor Technologies with Nanoholes-Provided High Areal Coverages and Their Application in Plasmonic-Enhanced Thin Film Photovoltaics

Zhaozhao Wang[1], Linfa Peng[1], Zhongqin Lin[1], Jun Ni[1,2], Peiyun Yi[1], Xinmin Lai [1], Xiaolong He[3], and Zeyu Lei[3]

[1]State Key Laboratory of Mechanical System and Vibration, Department of Mechanical Engineering, Shanghai Jiao Tong University, Shanghai, 200240, P.R. China.

[2]Department of Mechanical Engineering, University of Michigan, Ann Arbor, MI 48109-2125, USA.

[3]University of Michigan-Shanghai Jiao Tong University Joint Institute, National Key Laboratory of Nano/Micro Fabrication Technology, Shanghai Jiao Tong University, Shanghai 200240, P.R. China. Correspondence and requests for materials should be addressed to Linfa Peng (email: penglinfa@sjtu.edu.cn) or Jun Ni (email: junni@umich.edu).

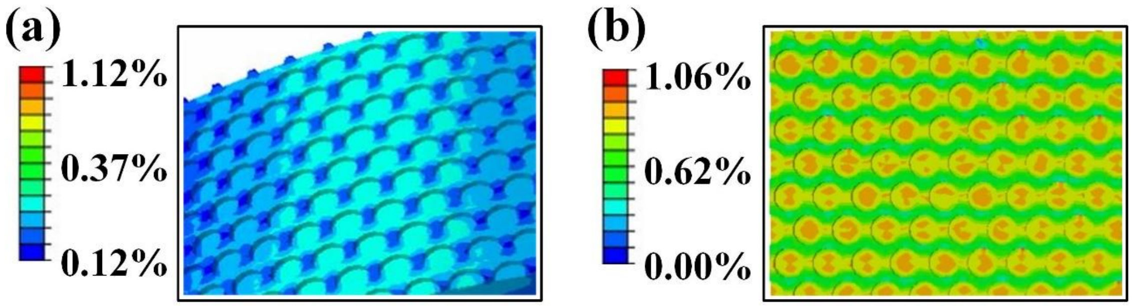

Figure S1. (a) Finite element modeling (FEM) of the patterned substrate bending with the curvature radius of 100 μm; (b) Finite element modeling (FEM) of the patterned substrate under applied uniaxial strain of 1.0%.

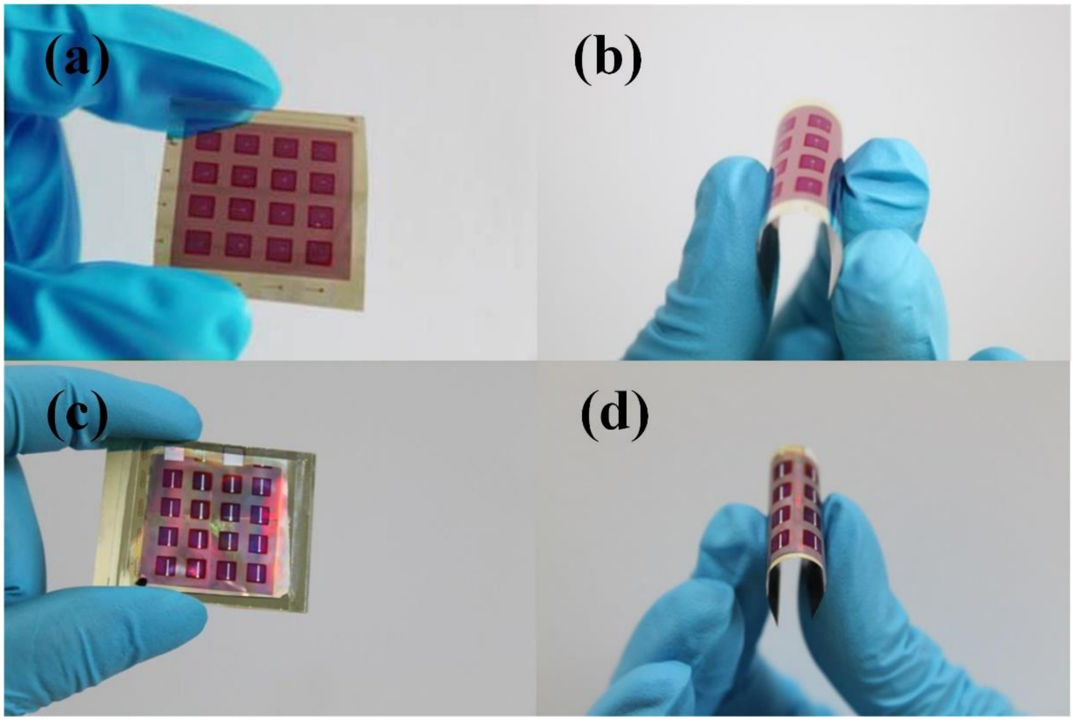

Figure S2. Photographs of a-Si:H solar cells (a) constructed on a flat PI film; (b) constructed on a flat PI film when curved; (c) constructed on a Patterned PI film; (d) constructed on a Patterned PI film when curved.

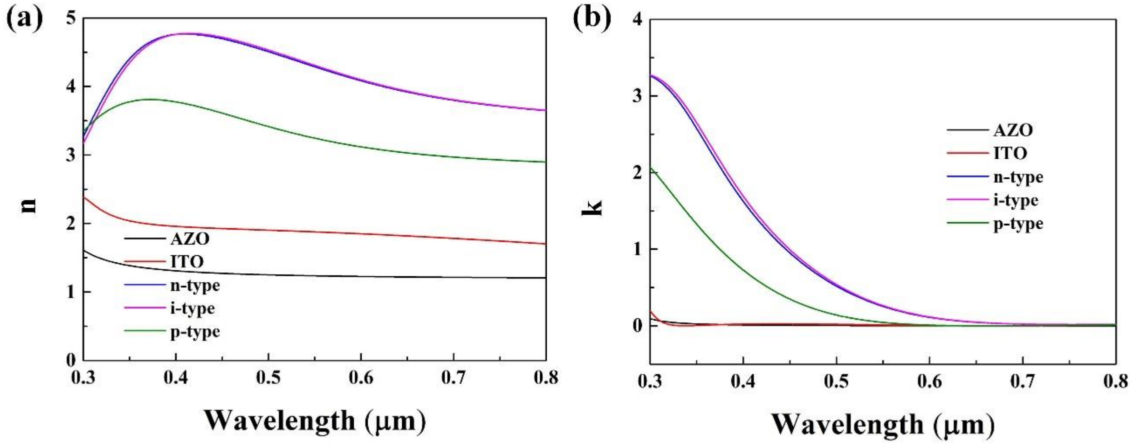

Figure S3. Refractive indices (a) and extinction coefficient (b) of ITO, AZO and n-i-p amorphous silicon layers respectively.
